# BASDAI versus ASDAS in evaluating axial involvement in patients with psoriatic arthritis: a pooled analysis of two phase 3 studies

**DOI:** 10.1093/rap/rkae058

**Published:** 2024-04-23

**Authors:** Xenofon Baraliakos, Dafna D Gladman, Soumya D Chakravarty, Cinty Gong, May Shawi, Emmanouil Rampakakis, Mitsumasa Kishimoto, Enrique R Soriano, Philip J Mease

**Affiliations:** Rheumazentrum Ruhrgebiet, Ruhr-University Bochum, Herne, Germany; Department of Medicine, University of Toronto, Schroeder Arthritis Institute; Krembil, Research Institute; Toronto Western Hospital, Toronto, ON, Canada; Immunology, Janssen Scientific Affairs, LLC, a Johnson & Johnson company, Horsham, PA, USA; Division of Rheumatology, Drexel University College of Medicine, Philadelphia, PA, USA; Immunology, Janssen Research & Development, LLC, a Johnson & Johnson company, Spring House, PA, USA; Immunology, Janssen Research & Development, LLC, a Johnson & Johnson company, Titusville, NJ, USA; Department of Pediatrics, McGill University, Montreal, Canada; Scientific Affairs, JSS Medical Research, Inc, Montreal, Canada; Department of Nephrology and Rheumatology, Kyorin University School of Medicine, Mitaka, Tokyo, Japan; Rheumatology Section, Internal Medicine Service, Department of Medicine, Hospital Italiano de Buenos Aires, Buenos Aires, Argentina; Department of Medicine, University Institute Hospital Italiano de Buenos Aires, Buenos Aires, Argentina; Rheumatology Research, Providence Swedish Medical Center, Seattle, WA, USA; University of Washington School of Medicine, Seattle, WA, USA

**Keywords:** spondyloarthropathies, psoriatic arthritis, diagnostic imaging, MRI, spine, clinical trials, BASDAI, ASDAS

## Abstract

**Objective:**

In the absence of axial psoriatic arthritis (axPsA)-specific tools, the BASDAI and Ankylosing Spondylitis Disease Activity Score (ASDAS) are used to assess axial symptoms in patients with PsA. Here, we assessed the performance of BASDAI and ASDAS in patients with PsA.

**Methods:**

Patients with active PsA in DISCOVER-1 and DISCOVER-2 (ClinicalTrials.gov: NCT03162796 and NCT03158285, respectively) with or without axPsA but with available baseline BASDAI information were analysed; those with investigator-identified axial symptoms and imaging-confirmed sacroiliitis comprised the axPsA cohort. Correlations between BASDAI/ASDAS and clinical variables were assessed with Pearson’s coefficient (*r*). Longitudinal effects of enthesitis (Leeds Enthesitis Index [LEI]), swollen joint count and presence versus absence of axPsA on BASDAI/ASDAS (normalized 0–10 scale) were analysed with mixed models for repeated measures.

**Results:**

At baseline in the axPsA (*n* = 312) and non-axPsA (*n* = 124) cohorts, BASDAI scores showed no or weak correlation with swollen joint count (0.18–0.20), tender joint count (0.12–0.29), LEI (–0.04 to 0.24) and physician global assessment (0.35–0.43); moderate correlation with fatigue (both −0.56); and strong correlation with patient global assessment of disease activity (0.62–0.69) and patient-reported pain (0.66–0.70). Similar correlations were observed for ASDAS. Axial involvement versus non-involvement was associated with higher BASDAI scores and ASDAS (all β ≥ 0.5), without differences between instruments; longitudinal associations between swollen joint count (β ≤ 0.06)/LEI (β ≤ 0.19) and BASDAI/ASDAS were clinically unimportant.

**Conclusion:**

BASDAI and ASDAS performed similarly in patients with active PsA and axial involvement, independent of peripheral disease involvement, supporting their performance in assessing axial disease activity.

**Trial registration:**

ClinicalTrials.gov, http://clinicaltrials.gov, NCT03162796 and NCT03158285.

Key messages

- In patients with active PsA, BASDAI scores and ASDAS correlated moderately-to-strongly with patient-reported outcomes.
- The BASDAI and ASDAS performed comparably in detecting changes in axial symptoms in PsA patients.
- Findings contribute to efforts to define axial disease and assess treatment outcomes in PsA patients.

## Introduction

PsA is a chronic, immune-mediated, inflammatory disease characterized by involvement across various clinical domains, such as peripheral arthritis, dactylitis, enthesitis and axial disease, in addition to skin and nail involvement [1, 2]. PsA with axial involvement or axial PsA (axPsA), is characterized by inflammation and associated structural changes of the spine and/or sacroiliac joints. The condition often manifests as back pain typical of inflammatory characteristics related to axial skeletal disorders [3]. Radiographic, molecular, genetic and biomarker data suggest that axPsA is distinct from axial spondyloarthritis (axSpA) [3, 4]. This distinction is also supported by studies showing improvements with ustekinumab, an IL-12/IL-23 inhibitor (i), in treating axial symptoms in patients with PsA [5], but not in the treatment of patients with radiographic or nonradiographic axSpA [6]. The humanized IL-23i risankizumab, which is approved for PsA, did not demonstrate efficacy in patients with radiographic axSpA [7]. Although not studied in patients with active axSpA, the fully human IL-23p19-subunit inhibitor guselkumab (Janssen Biotech, Horsham, PA, USA) [8], showed significant and meaningful improvements in symptoms of axial disease in *post hoc* analyses of PsA patients with axial involvement (identified by investigator-confirmed imaging) [9, 10].

Evaluation of patients with axPsA in clinical trials has been limited by the absence of standard criteria in diagnosing or classifying axial involvement in PsA patients as well as aspects of trial design and lack of axPsA-specific assessment tools [11]. The Axial Involvement of Psoriatic Arthritis Cohort (AXIS) study is being conducted to develop evidence-based consensus classification criteria and nomenclature for axPsA to facilitate the identification and examination of homogenous axPsA cohorts in the research setting [12]. In the absence of axPsA-specific assessment tools, the BASDAI and Ankylosing Spondylitis Disease Activity Score (ASDAS), originally developed to evaluate patients with radiographic axSpA, are typically utilized to evaluate axPsA disease activity [13, 14]. Although the BASDAI is used to assess axial involvement in patients with PsA, only one of its questions (question 2) is specific to spinal pain, assessing neck, back or hip pain [13]. In addition to spinal pain, the BASDAI provides a broad assessment of disease activity, with questions evaluating fatigue, peripheral joint pain, enthesitis and the duration and severity of morning stiffness; similar total scores have been observed in patients with PsA with or without axial disease [15, 16]. The ASDAS, which includes CRP level and three questions (spinal pain, peripheral joint pain and morning stiffness duration) from the BASDAI but does not include assessment of enthesitis and assigns less weight to peripheral disease activity, is believed by some to be more objective than the BASDAI [17].

The IL-23/IL-17 pathway is associated with the pathogenesis of PsA. IL-23 promotes maturation and pathogenicity of T helper (Th) 17 cells, resulting in the production of effector molecules, including IL-17A/F, IL-22 and TNFα [18]. Guselkumab demonstrated multidomain efficacy and a favourable safety profile in the phase 3 DISCOVER-1 and DISCOVER-2 studies of adults with active PsA [19, 20]. As mentioned, in *post hoc* pooled analyses of patients with PsA and imaging-confirmed sacroiliitis from DISCOVER-1 and DISCOVER-2, guselkumab improved axial symptoms versus placebo, as assessed by the BASDAI and ASDAS. Improvement with guselkumab treatment was observed as early as week 8 (the first time point assessed) and continued over 52 weeks in patients from both studies [9] and over 100 weeks in patients from the 2-year DISCOVER-2 study [10]. Utilizing BASDAI and ASDAS data from DISCOVER-1 and DISCOVER-2, the current *post hoc* analysis assessed the performance of these assessments in evaluating symptoms of axial involvement in patients with active PsA.

## Methods

### Study design and participants

The study design and patient eligibility criteria for DISCOVER-1 (clinicaltrials.gov Identifier: NCT03162796) and DISCOVER-2 (NCT03158285) have been described [19, 20]. In brief, these phase 3, randomized, double-blind, placebo-controlled trials evaluated the efficacy and safety of guselkumab in patients with active PsA despite standard therapies. In DISCOVER-1, patients with active PsA had a swollen joint count (SJC) ≥3, a tender joint count (TJC) ≥3 and CRP ≥0.3 mg/dl. In DISCOVER-2, active PsA was defined by SJC ≥5, TJC ≥5 and CRP ≥0.6 mg/dl. In DISCOVER-1, approximately 30% of enrolled patients received 1 or 2 prior TNFi; the remainder of DISCOVER-1 and all DISCOVER-2 patients were biologic-naive. Eligible patients were randomized 1:1:1 to guselkumab 100 mg every 4 weeks (Q4W); guselkumab 100 mg at week 0, week 4 and then every 8 weeks (Q8W); or placebo with crossover to guselkumab Q4W at week 24. Efficacy outcomes were assessed through week 52 and week 100 in DISCOVER-1 and DISCOVER-2, respectively. For this *post hoc* analysis, data through week 52 were pooled across the two trials with all treatment groups combined.

Among the 1120 randomized and treated patients in DISCOVER-1 and DISCOVER-2, patients were included in the axPsA cohort if they were identified by study investigators as having PsA with axial involvement (yes or no) and had sacroiliitis confirmed by a previous radiograph or magnetic resonance imaging (MRI; DISCOVER-1 and DISCOVER-2) or pelvic radiograph at screening (DISCOVER-2) (*n* = 312) [9]. All imaging was reviewed locally by the investigator [9]. Per protocol, the BASDAI questionnaire was intended to be completed only by patients identified by the investigator as having spondylitis. However, it was inadvertently also completed by 104 patients who were not identified by the investigator as having spondylitis at screening. For these 104 patients, imaging of the sacroiliac joints was not performed. These patients, along with 20 patients who were identified as having spondylitis but did not have investigator-confirmed imaging, constitute the non-axPsA cohort reported herein (*n* = 124). The remaining 684 patients from DISCOVER-1 and DISCOVER-2 were excluded from these analyses as they did not have an available BASDAI score.

DISCOVER-1 and DISCOVER-2 were conducted in accordance with the Declaration of Helsinki and Good Clinical Practice guidelines, and all participants provided written informed consent. The study protocols were approved by the governing ethical body at each study site (Sterling institutional review board approval numbers [US sites]: 5959C and 5910C).

### Assessments

This *post hoc* analysis assessed performance of the BASDAI and ASDAS in evaluating axial involvement in patients with active PsA. The BASDAI is a self-administered assessment of six symptoms measured on a visual analogue scale (VAS) between 0 and 10 [13]. Measured symptoms include fatigue, spinal pain, peripheral joint pain and swelling, pain at entheseal sites and severity and duration of morning stiffness. A modified version of the BASDAI (mBASDAI), which in this analysis excluded the questions related to peripheral joint pain and enthesitis, was also utilized to limit the impact of peripheral joint disease on the total score. The following formula was used to calculate the mBASDAI score: mBASDAI = ((Q1 + Q2) + ((Q5 + Q6)/2))/3. A separate modified version of the BASDAI excluding only the question related to peripheral joint pain has been reported to correlate with physician and patient global disease activity scores [21].

In DISCOVER-1 and DISCOVER-2, the ASDAS composite score utilized BASDAI components for spinal pain, peripheral joint pain/swelling and duration of morning stiffness (questions 2, 3 and 6 in the BASDAI, respectively); patient global assessment of disease activity (PtGA-Arthritis, VAS 0–10 cm); and CRP level (assessed by a central laboratory) [14]. All axPsA and non-axPsA patients had available serum CRP levels; thus, ASDAS was calculated for all patients with available BASDAI component scores and PtGA-Arthritis. A modified version of the ASDAS (mASDAS), excluding the same assessment of peripheral joint pain omitted from the mBASDAI, was also utilized. The mASDAS was calculated as:  0.12 × spinal pain + 0.06 × duration of morning stiffness + 0.11 × PtGA + 0.58 × ln (CRP + 1). Normalized ASDAS and mASDAS were calculated to balance the differences in scale among the dependent variables. Normalized ASDAS (based on a maximum ASDAS of ∼7 [22]; no arithmetic upper limit) was calculated as: 1.4286 × ASDAS; normalized mASDAS (based on a maximum mASDAS of ∼6.3) was calculated as: 1.5873 × mASDAS.

SJC (0–66 joints), TJC (0–68 joints), enthesitis (Leeds Enthesitis Index [LEI]) [23], fatigue (Functional Assessment of Chronic Illness Therapy [FACIT]–Fatigue) [24], physician global assessment (PhGA; VAS 0–10 cm), patient’s assessment of pain (VAS 0–10 cm) and PtGA were assessed in DISCOVER-1 and DISCOVER-2 as previously described [19, 20].

### Statistical analysis

Correlations between assessments of axial disease (BASDAI, mBASDAI, ASDAS or mASDAS) and other efficacy assessments (SJC, TJC, enthesitis, fatigue, PhGA, patient’s assessment of pain and PtGA) were evaluated at baseline (active disease) and week 24 (less active disease for some). Correlation was assessed with the Pearson’s correlation coefficient (*r*), separately among patients in the axPsA and non-axPsA cohorts. Very strong correlation was defined as a Pearson’s correlation coefficient ranging from 0.80 to 1.0, strong correlation as 0.60–0.79, moderate correlation as 0.40–0.59, weak correlation as 0.20–0.39 and very weak or no correlation as <0.20 [25].

The longitudinal impact of presence versus absence of axPsA symptoms was assessed using mixed models for repeated measures (MMRM) and included BASDAI, mBASDAI, normalized ASDAS or normalized mASDAS, each in separate models, over 52 weeks as the dependent variable; time, presence of axPsA and prior TNFi use (yes or no) as fixed factors; and LEI score and SJC as time-dependent covariates. For enthesitis, the impact of LEI score was assessed among patients with enthesitis at baseline also by employing MMRM, including BASDAI, mBASDAI, normalized ASDAS or normalized mASDAS, each in separate models, over 52 weeks as the dependent variable; time as a fixed factor; and LEI score and SJC as time-dependent covariates. The longitudinal impact of SJC was assessed among patients without enthesitis at baseline. In this analysis, the MMRM included BASDAI, mBASDAI, normalized ASDAS or normalized mASDAS, each in separate models, over 52 weeks as the dependent variable; time as a fixed factor; and SJC as a time-dependent covariate.

The standardized β was used to contrast the impact of axial disease on the different dependent variables. This measurement was chosen as this coefficient compares the strength of the effect on individual independent variables to the dependent variable. The β corresponds to the average incremental difference over 52 weeks of BASDAI/ASDAS values between patients in the axPsA versus non-axPsA cohorts and is equal to the difference in the estimated least-squares means of the two groups. A higher absolute value of the β coefficient corresponds with a stronger effect.

The discriminant performance of BASDAI, mBASDAI, ASDAS and mASDAS at baseline in assessing axial disease was evaluated using receiver operating characteristics (ROC) analysis and the associated area under the curve (AUC).

## Results

### Demographic and baseline characteristics

These *post hoc* analyses included 436 patients with active PsA (full analysis population). Of these, 312 patients comprised the axPsA cohort (289 of whom had available BASDAI data at baseline) and 124 patients comprised the non-axPsA cohort. Of these 124 non-axPsA patients, 20 patients were identified by the investigators as having peripheral arthritis with spondylitis but were retained in the non-axPsA cohort as they did not have imaging confirmation of sacroiliitis. Baseline demographic and clinical disease characteristics were generally similar among the pooled DISCOVER-1 and DISCOVER-2 patient population (*n* = 1120) and the axPsA and non-axPsA (data not shown) patient cohorts in this *post hoc* analysis; although, the axPsA cohort was characterized by somewhat higher proportions of patients who were male (61% *vs* 52%) and who had enthesitis (72% *vs* 65%) and dactylitis (50% *vs* 42%) and a higher mean CRP level (2.5 *vs* 1.8 mg/dl) relative to the overall pooled study population (Table 1).

**Table 1.** Demographic and baseline characteristics of pooled DISCOVER-1 and DISCOVER-2 patients

**Parameter, mean (s.d.)** ^a^	All patients *n* = 1120	**axPsA cohort** ^b^ ***n* = 312**
Age, years	46.6 (11.7)	45.1 (11.2)
Male, %	52	61
BMI, kg/m^2^	29.2 (6.1)	28.1 (6.3)
PsA duration, years	5.9 (6.1)	5.7 (5.8)
SJC (0–66)	11.4 (7.4)	11.3 (7.3)
TJC (0–68)	20.6 (13.3)	22.0 (14.0)
CRP, median (Q1–Q3), mg/dl	0.9 (0.5–2.2)	1.5 (0.7–3.0)
PASI (0–72)	9.5 (10.6)	10.8 (11.4)
Patients with enthesitis, %	65	72
LEI score (1–6)	2.8 (1.6)	2.8 (1.7)
Patients with dactylitis, %	42	50
Dactylitis score (1–60)	8.2 (9.6)	8.2 (9.1)
FACIT-Fatigue	29.9 (10.0)	28.6 (9.3)
PhGA (VAS, 1–10 cm)	6.5 (1.6)	6.7 (1.6)
Patient-reported pain (VAS, 1–10 cm)	6.1 (2.0)	6.3 (1.8)
PtGA (VAS, 1–10 cm)	6.7 (2.0)	6.9 (1.9)
BASDAI	–	6.5 (1.7)^c^
ASDAS	–	3.9 (0.9)^c^

a Data are presented as mean (s.d.) unless stated otherwise.

b Patients with active PsA and investigator-confirmed axial involvement and sacroiliitis documented by a previous radiograph or magnetic resonance imaging (DISCOVER-1 and DISCOVER-2) or pelvic radiograph (DISCOVER-2) at screening. Availability of BASDAI and ASDAS values at baseline was not a criterion of inclusion in the axPsA cohort.

c *n* = 289 with available BASDAI and ASDAS values.

axPsA: axial PsA; LEI: Leeds Enthesitis Index; PASI: Psoriasis Area and Severity Index; PhGA: physician global assessment; PtGA: patient global assessment; SJC: swollen joint count; TJC: tender joint count.

### Correlation of BASDAI and ASDAS with clinical variables at baseline and week 24

When assessing correlations with BASDAI at baseline among patients in the axPsA and non-axPsA cohorts with available BASDAI data, weak correlations (*r *≤* *0.39) were observed with SJC, TJC and LEI; weak/moderate correlation with FACIT-Fatigue (*r *=* *−0.56 in both cohorts) and PhGA (*r *=* *0.35–0.43); and strong correlation with patient-reported pain (*r *=* *0.66–0.70) and PtGA (*r *=* *0.62–0.69; Table 2). Analogous correlations were observed between ASDAS and these clinical variables at baseline. Similar relationships were also observed for BASDAI, ASDAS, mBASDAI (excluding the peripheral joint pain and enthesitis questions) and mASDAS (excluding the peripheral joint pain question) and clinical variables at week 24; although, the *r* values tended to be higher for most correlations than those observed at baseline, demonstrating a stronger association when mean levels of disease activity are lower (Table 2).

**Table 2.** Correlation of BASDAI and ASDAS With Clinical Variables at Baseline and Week 24 Across All Treatment Groups

	axPsA cohort (*n* = 312)				non-axPsA cohort^a^ (*n* = 124)			
**Baseline**	**BASDAI**	**mBASDAI**	**ASDAS**	**mASDAS**	**BASDAI**	**mBASDAI**	**ASDAS**	**mASDAS**
SJC	0.20*	0.17*	0.18*	0.17*	0.18*	0.16	0.19*	0.18
TJC	0.29*	0.27*	0.21*	0.20*	0.12	0.11	0.14	0.14
LEI score	0.24*	0.23*	0.17*	0.16*	–0.04	–0.02	0.07	0.08
FACIT-Fatigue^b^	–0.56*	–0.55*	–0.37*	–0.34*	–0.56*	–0.56*	–0.50*	–0.47*
PhGA	0.35*	0.33*	0.44*	0.43*	0.43*	0.41*	0.44*	0.43*
Patient-reported pain	0.66*	0.61*	0.62*	0.58*	0.70*	0.67*	0.70*	0.66*
PtGA	0.69*	0.64*	0.62*	0.59*	0.62*	0.59*	0.60*	0.57*
**Week 24**	**BASDAI**	**mBASDAI**	**ASDAS**	**mASDAS**	**BASDAI**	**mBASDAI**	**ASDAS**	**mASDAS**
SJC	0.38*	0.36*	0.39*	0.38*	0.07	0.05	0.06	0.05
TJC	0.45*	0.43*	0.40*	0.38*	0.32*	0.62*	0.37*	0.40*
LEI score	0.27*	0.27*	0.23*	0.21*	0.25	0.20	0.23	0.22
FACIT-Fatigue^b^	–0.66*	–0.68*	–0.53*	–0.50*	–0.55*	–0.56*	–0.57*	–0.60*
PhGA	0.53*	0.53*	0.58*	0.58*	0.30*	0.26	0.39*	0.41*
Patient-reported pain	0.89*	0.85*	0.81*	0.77*	0.89*	0.85*	0.80*	0.72*
PtGA	0.88*	0.86*	0.79*	0.74*	0.92*	0.89*	0.84*	0.77*

a Per study protocol, the BASDAI questionnaire was to be completed only by patients classified with primary PsA subtype of “peripheral arthritis with spondylitis” confirmed by radiograph or MRI (axPsA cohort); however, it was inadvertently completed by some patients classified as having a different primary PsA subtype or without imaging confirmation (non-axPsA cohort).

b Higher scores indicate worse fatigue.

* Statistically significant *P* <0.05.

Pearson’s correlation (*r*) - Very weak/no correlation: *r* <0.20; Weak: *r* = 0.20-0.39; Moderate: *r* = 0.40-0.59; Strong: *r* = 0.60-0.79; Very strong: *r* = 0.80-1.00

ASDAS, Ankylosing Spondylitis Disease Activity Score; axPsA, axial PsA; FACIT, Functional Assessment of Chronic Illness Therapy; LEI, Leeds Enthesitis Index; mASDAS, modified ASDAS; mBASDAI, modified BASDAI; PhGA, physician global assessment; PtGA, patient global assessment of disease activity.

### Longitudinal effects of axial involvement on BASDAI and ASDAS 

**Figure 1.**
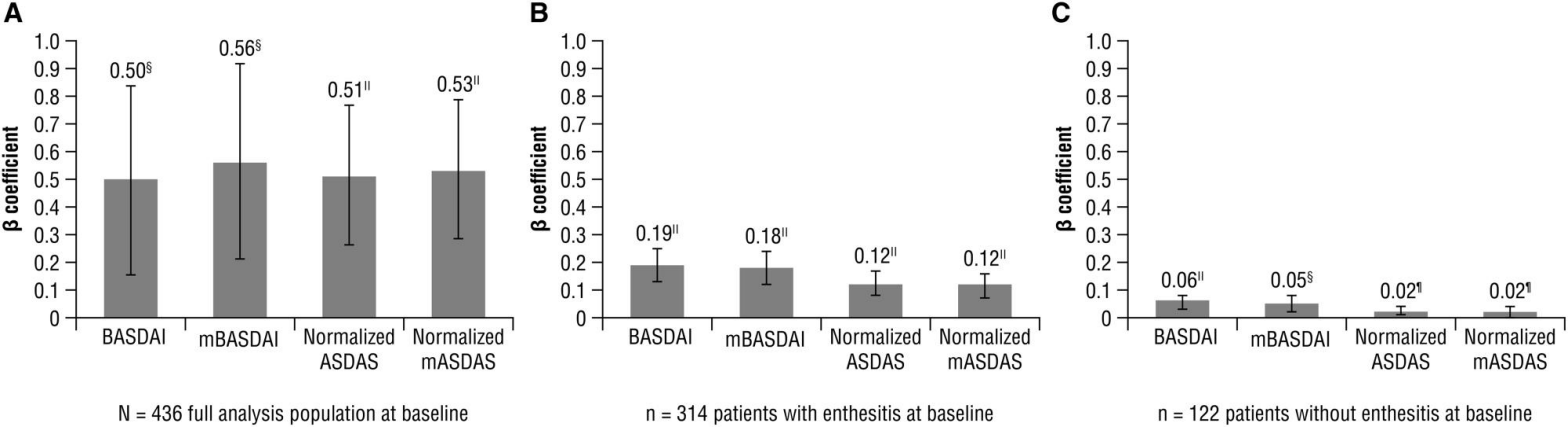
Associations between BASDAI/ASDAS over 52 weeks and (A) axial involvement,^a^ (B) LEI score,^b^ and (C) SJC.^c^ Impact evaluated with MMRM including BASDAI/mBASDAI/normalized ASDAS/normalized mASDAS (separately) over 52 weeks as the dependent variable and ^a^time, imaging-confirmed axial involvement, and prior TNFi use as fixed factors, and LEI score and SJC as time-dependent covariates; ^b^time as a fixed factor, and LEI score and SJC as time-dependent covariates; and ^c^time as a fixed factor, and SJC as time-dependent covariate. ^¶^*P* ≤0.05, ^§^*P* ≤0.01, ^‖^*P* ≤0.001. ASDAS: Ankylosing Spondylitis Disease Activity Score; LEI: Leeds Enthesitis Index; mASDAS: modified Ankylosing Spondylitis Disease Activity Score; mBASDAI: modified BASDAI; MMRM: mixed models for repeated measures; SJC: swollen joint count

In the full analysis population, imaging-confirmed axial involvement (axPsA cohort), compared with no confirmed axial involvement (non-axPsA cohort), was associated with significantly greater BASDAI (β = 0.50), mBASDAI (β = 0.56), normalized ASDAS (β = 0.51) and mASDAS (β = 0.53) scores. No apparent differences in performance were observed between instruments (Fig. 1A).

### Longitudinal effects of enthesitis and SJC on BASDAI and ASDAS

Among the 314 patients with enthesitis at baseline, statistically significant associations were observed between LEI score and BASDAI, mBASDAI, normalized ASDAS and normalized mASDAS (Fig. 1B). The low standardized β coefficients (all ≤0.19) observed indicate a weak relationship between enthesitis and BASDAI/ASDAS.

Among the 122 patients without enthesitis at baseline, SJC showed statistically significant associations with BASDAI, mBASDAI, normalized ASDAS and normalized mASDAS (Fig. 1C). The low standardized β coefficients (all ≤0.06) indicated a weak relationship between SJC and BASDAI/ASDAS.

### Discriminant performance

**Figure 2.**
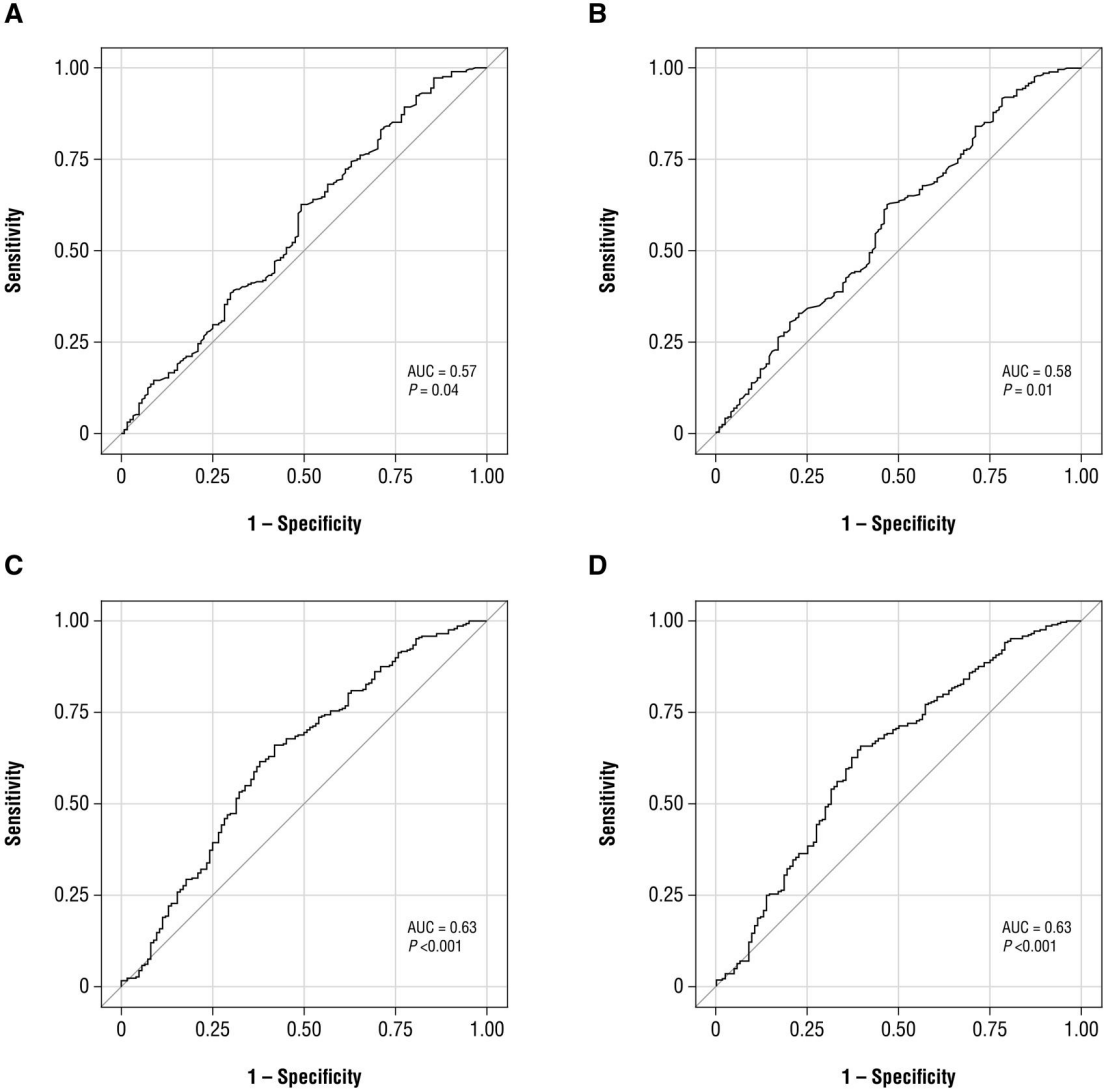
Baseline (A) BASDAI, (B) mBASDAI, (C) ASDAS, and (D) mASDAS performance in assessing axial disease. *n*** **=** **289 patients in the axPsA cohort (with available baseline BASDAI score); *n*** **=** **124 patients in the non-axPsA cohort. ASDAS: Ankylosing Spondylitis Disease Activity Score; AUC: area under the curve; axPsA: axial PsA; mASDAS: modified Ankylosing Spondylitis Disease Activity Score; mBASDAI: modified BASDAI

The BASDAI, mBASDAI, ASDAS and mASDAS demonstrated similar discriminant performance in assessing axial disease at baseline based on ROC analyses. The AUC (standard error) values were 0.57 (0.03 [lower area = 0.50 and upper area = 0.63], *P *=* *0.04) for BASDAI and 0.58 (0.03 [lower area = 0.52 and upper area = 0.64], *P *=* *0.01) for mBASDAI. The normalized ASDAS and mASDAS demonstrated an AUC (standard error) of 0.63 (0.03 [lower area = 0.57 and upper area = 0.69], *P *<* *0.001) and 0.63 (0.03 [lower area = 0.57 and upper area = 0.69], *P *<* *0.001), respectively (Fig. 2).

## Discussion

This *post hoc* analysis assessing the BASDAI and ASDAS in axPsA and non-axPsA cohorts showed that these instruments correlated similarly with clinical variables and performed comparably in their ability to classify and discern changes in axial symptoms in patients with active PsA. Associations seen between axial involvement and BASDAI score, normalized ASDAS and mASDAS support the use of these composite indices in PsA patients with axial involvement in the absence of PsA-specific assessment tools for axial disease. Modified versions of the BASDAI and ASDAS were also used in these analyses to minimize the influence of improvements in peripheral joint pain and enthesitis in these composite scores.

The discriminant performance of BASDAI and ASDAS were analogous in evaluating baseline axial disease. These instruments also performed similarly in assessing associations between axial involvement and clinical measures (LEI score and SJC). The results of the current analysis reinforce findings of previous analyses from DISCOVER-1 and DISCOVER-2, wherein both instruments were able to detect treatment effects [9, 10]. In these prior *post hoc* analyses in patients from this axial cohort, guselkumab-treated patients achieved significantly greater mean improvements in the BASDAI and ASDAS compared with those receiving placebo. Mean improvements in total BASDAI and ASDAS were maintained through up to 2 years, with reductions noted across all six BASDAI components [9, 10].

In the current analysis, patients in the axPsA cohort demonstrated improvements in axial symptoms when assessed by the BASDAI; however, question 2 is the only question in the instrument that directly assessed axial-related symptoms [13]. This limitation has informed the evaluation of other instruments, like the ASDAS, to discern the correlation of these tools with clinical variables associated with axPsA. Other efforts to compare the discriminative ability of the BASDAI and ASDAS as tools to measure disease activity in patients with axPsA showed that both instruments have a similar ability to distinguish between high and low disease activity [21, 26]. Consistent with the strong correlations observed herein between baseline BASDAI score and patient-related pain/PtGA scores in both axPsA and non-axPsA cohorts, a prior study also showed patient-reported disease activity measures tended to have a stronger correlation with BASDAI or ASDAS scores than physician-reported measures [21]. These findings are not unexpected given that both instruments rely on patient-reported outcomes versus objective clinical assessments, with the exception of the inclusion of CRP level in the ASDAS [13, 14].

Data suggest that axial involvement can occur in 25–70% of patients with PsA, depending on patient selection criteria [3, 12]. The axPsA cohort of the current *post hoc* analysis (*n* = 312) comprised 28% of the total pooled DISCOVER-1 and DISCOVER-2 population (*n* = 1120). As investigator-identified axial involvement was confirmed only by the presence of sacroiliitis and patients were not prospectively evaluated for the presence of spinal inflammation, it is therefore possible that patients were misclassified as having versus not having axial involvement. Ongoing studies, like AXIS, aim to evaluate clinical and imaging parameters typical of axial involvement in patients with PsA to develop unified guidelines that would allow for the enrolment of a standardized cohort of patients with axial involvement in clinical trials [12]. Concurrently, the phase 4, randomized, double-blinded, placebo-controlled STAR study aims to evaluate the efficacy and safety of treatment with guselkumab in patients with PsA and MRI-confirmed axial inflammation [27]. In contrast to the DISCOVER studies, MRIs of sacroiliac joints and the spine will be evaluated by blinded central readers as an eligibility criterion, using methodologies specifically created to assess axial inflammation. Both AXIS and STAR, in addition to the findings reported herein, will help in the efforts to define disease and assess treatment outcomes in patients with PsA and axial involvement.

A strength of the current analyses was inclusion of a relatively large number of PsA patients with axial involvement who had available BASDAI and ASDAS measures; although, the number of patients without axial involvement with BASDAI and ASDAS data available for comparison was relatively small. As specified in the study protocols, the BASDAI was to be administered only to patients classified as having spondylitis with peripheral arthritis, but some patients classified as having other PsA subtypes inadvertently completed the questionnaire, comprising the non-axPsA cohort in the present analyses.

Evidence to confirm the presence of axial disease is limited. In DISCOVER-1 and DISCOVER-2, patients with axial involvement were identified by the investigator and imaging confirmation was restricted to the evaluation of the sacroiliac joints and was reviewed locally by the investigator without verification by central readers. This approach is limited by a higher degree of variability relative to assessment by central readers [28]. Additionally, because DISCOVER-1 and DISCOVER-2 patients were not objectively assessed for spinal inflammation, some participants in the non-axPsA cohort may have been misclassified as not having axial involvement.

Accepted imaging techniques for verification of sacroiliitis prior to screening or at baseline varied between the DISCOVER studies (ie, prior radiograph or MRI for DISCOVER-1 and DISCOVER-2; pelvic radiograph at screening for DISCOVER-2). Furthermore, without obtaining imaging of the sacroiliac joints for all patients, it is unclear whether patients in the non-axPsA cohort were indeed unaffected by axial disease, including the 20 patients who were identified by the investigator as having axial involvement, but did not have imaging confirmation by either technique. Lastly, the *post hoc* nature of the analyses may limit the interpretation of these results. Despite these caveats, the data reported herein further our understanding of assessing treatment outcomes in patients with PsA and axial involvement.

In conclusion, the results of this *post hoc* analysis suggest that both BASDAI and ASDAS may perform comparably in assessing axial involvement in patients with PsA in a clinical setting. Peripheral disease involvement did not appear to affect the utility of BASDAI and ASDAS for the assessment of axPsA.

## Data Availability

The data sharing policy of Janssen Pharmaceutical Companies of Johnson & Johnson is available at https://www.janssen.com/clinical-trials/transparency. As noted on this site, requests for access to the study data can be submitted through the Yale Open Data Access (YODA) Project site at http://yoda.yale.edu.
